# Quantification of Coupled Stiffness and Fiber Orientation Remodeling in Hypertensive Rat Right-Ventricular Myocardium Using 3D Ultrasound Speckle Tracking with Biaxial Testing

**DOI:** 10.1371/journal.pone.0165320

**Published:** 2016-10-25

**Authors:** Dae Woo Park, Andrea Sebastiani, Choon Hwai Yap, Marc A. Simon, Kang Kim

**Affiliations:** 1 Center for Ultrasound Molecular Imaging and Therapeutics, Department of Medicine, University of Pittsburgh School of Medicine, Pittsburgh, Pennsylvania, United States of America; 2 Heart and Vascular Institute, University of Pittsburgh Medical Center (UPMC), Pittsburgh, Pennsylvania, United States of America; 3 Department of Biomedical Engineering, National University of Singapore, Singapore; 4 Department of Bioengineering, University of Pittsburgh School of Engineering, Pittsburgh, Pennsylvania, United States of America; 5 McGowan Institute for Regenerative Medicine, University of Pittsburgh and UPMC, Pittsburgh, Pennsylvania, United States of America; 6 Heart, Lung, and Blood Vascular Medicine Institute, University of Pittsburgh, Pittsburgh, Pennsylvania, United States of America; Semmelweis Egyetem, HUNGARY

## Abstract

Mechanical and structural changes of right ventricular (RV) in response to pulmonary hypertension (PH) are inadequately understood. While current standard biaxial testing provides information on the mechanical behavior of RV tissues using surface markers, it is unable to fully assess structural and mechanical properties across the full tissue thickness. In this study, the mechanical and structural properties of normotensive and pulmonary hypertension right ventricular (PHRV) myocardium through its full thickness were examined using mechanical testing combined with 3D ultrasound speckle tracking (3D-UST). RV pressure overload was induced in Sprague–Dawley rats by pulmonary artery (PA) banding. The second Piola–Kirchhoff stress tensors and Green-Lagrangian strain tensors were computed in the RV myocardium using the biaxial testing combined with 3D-UST. A previously established non-linear curve-fitting algorithm was applied to fit experimental data to a Strain Energy Function (SEF) for computation of myofiber orientation. The fiber orientations obtained by the biaxial testing with 3D-UST compared well with the fiber orientations computed from the histology. In addition, the re-orientation of myofiber in the right ventricular free wall (RVFW) along longitudinal direction (apex-to-outflow-tract direction) was noticeable in response to PH. For normotensive RVFW samples, the average fiber orientation angles obtained by 3D-UST with biaxial test spiraled from 20° at the endo-cardium to -42° at the epi-cardium (Δ = 62°). For PHRV samples, the average fiber orientation angles obtained by 3D-UST with biaxial test had much less spiral across tissue thickness: 3° at endo-cardium to -7° at epi-cardium (Δ = 10°, P<0.005 compared to normotensive).

## Introduction

Right ventricular (RV) failure is a major cause of death for patients with pulmonary hypertension (PH), with high morbidity and mortality [1, 2]. The structural and functional changes associated with the RV remodeling process lead to ventricular failure [3]. However, the underlying mechanical behavior of the remodeled RV in PH on a tissue level has not been fully understood and could provide more insight into diagnosis and treatment [4, 5]. The RV tissues are known to have a complex architecture [4, 5, 6] and complex mechanical responses to loads [7]. Understanding their mechanical and structural information during the remodeling and failure of the RV myocardium will significantly aid in treatment approach for the PH and management of their mechanical failures [8].

The current standard method for measuring the passive mechanical properties of biological samples utilizes biaxial loading and strain measurements [9–11]. The strains of the tissue are usually measured by visually tracking markers (VTM) placed on the surface of the samples and imaged via conventional cameras [9]. Using the biaxial mechanical testing with VTM, the tissue constitutive models (stress-strain relation) have been established from animal RV tissues [10, 11] and the changes of mechanical properties in response to PH have been measured [12]. However, the current biaxial mechanical testing with VTM computes mechanical properties based on assumptions that RV tissues are homogeneous [13] and generate uniform strains in response to loads across tissue thickness [9–12]. It should be noted that most RV tissues are heterogeneous and strains in the tissues therefore are not uniform [13]. Assessing their mechanical behavior coupled with transmural structural information such as fiber orientation will augment understanding of the complex mechanical behavior of heterogeneous RV tissues. In recent study by Hill et al [12], both mechanical and microstructural changes in rat RV free wall samples in response to RV pressure overload were investigated using standard biaxial testing (using VTM). This study found an increase in the intrinsic stiffness of the remodeled RV myocardium predominately in the longitudinal direction (apex-to-outflow-tract direction) associated with re-orientation of myo- and collagen fibers from an endo- to epi-cardial spiral pattern to a nearly homogenous longitudinal orientation.

One inherent limitation of standard biaxial testing with VTM is that it provides only two-dimensional (2D) strains on the surface of samples and cannot provide strains beneath the surface, coupled with three-dimensional (3D) transmural structures such as myocardium fiber orientations. Thus, the current biaxial mechanical testing with VTM technique can only be applied to thin tissue samples for which reconstructed mechanical properties assume a 2D tissue model. The lack of 3D transmural strain information inevitably limits the biaxial mechanical testing with VTM especially for thickened right ventricular free wall (RVFW), resulting in inaccurate interpretation of the RVFW remodeling process, which varies through thickness.

The 3D ultrasound speckle tracking (3D-UST) [14] can overcome this limitation since it can assess the 3D deformations non-invasively in a 3D volume sample that is optically opaque and thick [15]. In addition, the biaxial testing combined with 3D-UST allows for concurrent assessment of coupled mechanical and structural characteristics of thick tissues. With 3D tissue displacements and boundary stress information, spatially-varying fiber orientations can be computed from the Strain Energy Function (SEF) [15].

In our previous study [15], a rat left ventricular (LV) myocardium was tested with a biaxial mechanical testing, while it was concurrently 3D scanned with high-frequency ultrasound. The SEF and spatially varying fiber orientations were computed from stresses and strains of a left ventricular free wall (LVFW) sample using an iterative non-linear curve-fitting algorithm. The accuracy of the non-linear curve-fitting algorithm for fiber orientation computation was evaluated using in silico finite element analysis in the same study.

The purpose of this study is, using the biaxial testing combined with 3D-UST, to elucidate the changes of mechanical properties and remodeling processes of fiber orientation in RV myocardium that occur in response to chronic pressure overload in an animal model of PH. A pulmonary artery (PA) banding model was utilized to generate the PH condition in a rat. The biaxial testing combined with 3D-UST was performed to obtain mechanical properties of RVFW and quantify the fiber orientation remodeling. The mechanical properties obtained by the biaxial testing combined with 3D-UST were compared with those measured by the biaxial testing with VTM. The fiber orientations obtained by the biaxial testing combined with 3D-UST were compared with those computed from histology.

## Materials and Methods

### Animal preparation

The experimental protocol of this study was approved by the University of Pittsburgh Institutional Animal Care and Use Committees. A total of 15 male Sprague–Dawley rats, 8 weeks old at the start of the experiment, were used in this study. Of the 15 total rats, RV pressure overload was induced surgically by restriction of the pulmonary artery on 6 animals. The PA banding was kept for 3 weeks for all 6 animals to generate a chronic pressure overload.

### Pulmonary artery banding procedure

Procedure was performed as we have previously described [12]. Briefly, animals were anesthetized with 5% isoflurane and placed on a heated table to maintain a core temperature of 37°C, then a lateral thoracotomy was performed and a surgical clip was placed around the PA to generate a uniform RV pressure of 45–50 mmHg (approximately the diameter of a 27-gauge needle). After chest closure, the animals were extubated and observed for 2 hours. The animals were then monitored per standard practice for 3 weeks.

### Sample preparation

Sample preparation was performed as we have previously described [12, 16]. Briefly, after anaesthetization with 5% isoflurane, a midline thoracotomy was performed and the heart was excised. RVFW was isolated and adhesive markers were attached on the epi-cardium. The apical and outflow tract locations were marked in order to maintain proper tissue orientation (Y direction: along the axis from the outflow tract to the apex; X direction perpendicular to this). Sample size was approximately 7х7 mm and 3 mm thick. The samples were submerged in a saline bath at room temperature of around 25°C for the biaxial testing combined with 3D-UST.

### Biaxial mechanical testing and ultrasound scanning

**Fig 1:**
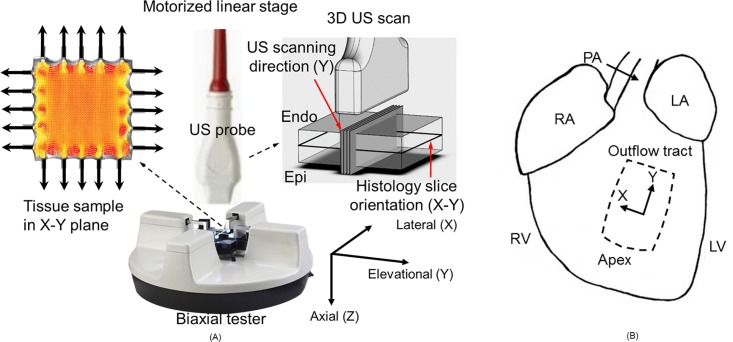
(A) Experimental setup for biaxial testing combined with 3D ultrasound scanning. X: lateral, Y: elevational (scanning), Z: axial (ultrasound propagation) direction of the ultrasound transducer. Note that the 3D speckle tracking and the displacement/strain estimation follow this coordinate. (B) The excised section of right ventricular free wall (RVFW) for biaxial testing. X: cross-preferred (circumferential) direction; Y: apex-to-outflow-tract (longitudinal) direction. Note that the X and Y in ultrasound transducer coordinate and the RVFW sample coordinate match respectively.

The biaxial testing and ultrasound scanning were performed using a procedure based on a previous study [15]. Experimental setup for biaxial testing combined with 3D ultrasound scanning is shown in Fig 1A. A commercial biaxial tester (BioTester, CellScale, Waterloo, ON, Canada) was used with simultaneous ultrasound imaging via a 30-MHz linear array transducer (MS400, VisualSonics, Toronto, ON, Canada) connected to a high-frequency ultrasound system (Vevo 2100, VisualSonics). Each B-scan cross-sectional image recorded had 784 axial samples over a distance of 12 mm and 444 lateral scan lines over a distance of 13.36 mm. The transducer has an axial resolution of 50 μm and lateral resolution of 110 μm. The linear array transducer was mounted onto a linear translational stage (Vevo Integrated Rail System, VisualSonics) to scan the elevational direction for 3D volumetric ultrasound images. A total of 39 cross-sectional imaging planes spaced by 0.102 mm were acquired.

Fig 1B shows the location and the orientation of the excised section of RVFW for biaxial testing; X: cross-preferred direction; Y: apex-to-outflow-tract direction. The sample was mounted onto the biaxial tester via small metal hooks such that the apex-base axis of the myocardium was aligned with the elevational axis (Y) of the ultrasound-biaxial experimental setup, the medial-lateral axis of the myocardium was aligned with the lateral axis (X) of the setup, while the trans-luminal axis of the myocardium was aligned with the axial axis (Z) of the setup.

After applying a mild preload of 3 mN, preconditioning was performed by stretching with strain rate of 1%/s from this initial condition to the highest stretch level for three cycles. Since each 3D ultrasound volume scan took approximately 4 sec and a quasi-static biaxial testing protocol was necessary, the sample was stretched to consecutively higher stretch levels with strain rate of 1%/s and the sample was held stationary for 6 sec at each stretch level during ultrasound imaging. Between stretches, the sample was maintained to the stretch-free state for 6 sec to alleviate stress relaxation effects. The biaxial test was performed twice on the same sample using different loading conditions based on previous established dual-loading protocol [15] to facilitate fitting of a material model to the ultrasound-biaxial test data. Note that the peak stretch level was set to be within the range of cardiac strain (12%–22%) so that the mechanical properties and fiber orientations are not altered after biaxial testing [17].

The load cell measured forces that were applied to the sample in the two stretch axes and the diagonals of the stress tensor were computed through dividing by the cross-sectional areas. The cross-sectional areas were measured from ultrasound images at a reference stretch condition which has zero strain at a load of 0.5 mN. The stresses at different conditions were computed by updating the stress at the reference condition with the stretch ratios accounting for area changes and with the measured force.

### Post-processing

The 3D-UST was performed following the same procedure as previously reported [15]. Briefly, the ultrasound in-phase and quadrature data collected were converted to radio frequency (RF) data by using standard quadrature sampling algorithms. The 3D-UST [14] was applied to 3D RF volume data to obtain 3D strain tensors, with a 3D kernel of 0.11 mm (Z) by 0.21 mm (X) by 0.30 mm (Y) based on the ultrasound speckle volume.

The 3D displacements between consecutive frames were accumulated to obtain the Lagrangian displacements for every frame, all of which were referenced to the pre-stretch condition. For the kinematic analysis, we assumed that RV samples were stationary with force equilibrium since the samples were held stationary for 6 sec at each stretch level during ultrasound imaging. The deformational gradient tensors and the right Cauchy–Green deformation tensors were calculated from the Lagrangian displacements. Eventually, the Green Lagrangian strains in longitudinal direction and circumferential direction were computed using the right Cauchy–Green deformation tensors[18]. The normal strains (E_XX_) in circumferential direction and (E_YY_) in longitudinal direction were determined using following equations.

$$F=\frac{dx}{dX}$$ (1)

$$C=F^{T}F$$ (2)

$$B={F∙F}^{T}$$ (3)

$$E=\frac{1}{2}(C-I)$$ (4)

where *X* is the reference coordinate, *x* is the deformed coordinate, **F** is the deformational gradient tensor, **C** is the right Cauchy–Green deformation tensor, **B** is the left Cauchy–Green deformation tensor, **E** is the Green Lagrangian strain and I is the identity matrix.

The mechanical property was modelled with a SEF (W) established by previous investigators [19], given by:

$$W=c_{1}{\left(\alpha -1\right)}^{2}+c_{2}{\left(\alpha -1\right)}^{3}+c_{3}(I_{1}-3){+c}_{4}(I_{1}-3)(\alpha -1)+c_{5}{\left(I_{1}-3\right)}^{2},$$ (5)

where *c*_1_–*c*_5_ are the SEF coefficients, *I*_1_ is the first invariant of the strain tensor, and *α* is the square root of the forth invariant of the strain tensor:

$$I_{1}=trC,$$ (6)

$$\alpha^{2}=N∙C∙N,$$ (7)

where N is the unit vector in the orientation of the embedded fiber. This SEF will result in a constitutive relationship as follows:

$$\sigma =-pI+2\frac{dW}{dI_{1}}B+\frac{1}{2\alpha }\frac{dW}{d\alpha }F{∙\text{N}⊗\text{N}∙F}^{T},$$ (8)

where **σ** is the Cauchy stress, and *p* is the Lagrangian multiplier that enforces incompressibility. The SEF coefficients were computed from the strain and stress tensors through an iterative non-linear curve fitting method that minimizes the sum-squared-error of the stress tensor diagonals according to previously established techniques [15].

### Validation of mechanical properties using biaxial testing with visual tracking markers

The mechanical properties of the RV samples obtained by biaxial testing with 3D-UST were compared with the mechanical properties of the same RV samples obtained by conventional biaxial testing with VTM. The biaxial testing with VTM was performed following the same procedure as previously reported [12]. Four graphite markers were attached to the epicardial surface of the central third of the RVFW specimens. From the marker positions, the deformation gradient tensor and the Green-Lagrangian strain tensor were calculated for each loading cycle using standard methods [12]. Since 3D-UST has some limitations in computing tissue displacements on the sample surface [14], the Green-Lagrange strains 0.11 mm below the tissue surface, which was adjusted based on actual 3D-UST kernel size, were estimated. The normal strains (E_XX_) in circumferential direction and (E_YY_) in longitudinal direction obtained by 3D-UST were compared with those obtained by VTM at each stress level using Mann-Whitney test. The Bland-Altman analysis was performed to further examine the relationship between the 3D-UST and VTM.

### Fiber orientation quantification with histology

After the biaxial testing, the same rat RVFW sample was fixed in formalin and dyed with standard Masson trichrome stain to obtain fiber orientations. A total of 9 slices with an incremental step of 300 μm were taken from each RVFW sample. The stained slides were digitally scanned with Nikon Super Coolscan 9000 ED (Nikon, Melville, NY, USA) at a 6.3-mm pixel resolution and analyzed with fast Fourier transformation using custom-written MATLAB (The Mathworks, Natick, MA, USA) programs at multiple points on the lateral-elevational plane. The fiber orientations were computed for each of 9 slices throughout the entire thickness (from endo- to epi-cardium). The fiber orientation angles obtained by histology were compared with those computed by 3D-UST biaxial experiments for the 9 slices using Mann-Whitney test. Note that in the ultrasound images, the 9 axial depths corresponding to 9 slices in histology samples were carefully chosen from the middle section of RVFW sample for comparison. In addition, the amount change in fiber orientation angles obtained by 3D-UST biaxial experiments from endo- to epi-cardium was compared between normotensive RV samples and PHRV samples using Mann-Whitney test.

## Results

**Fig 2:**
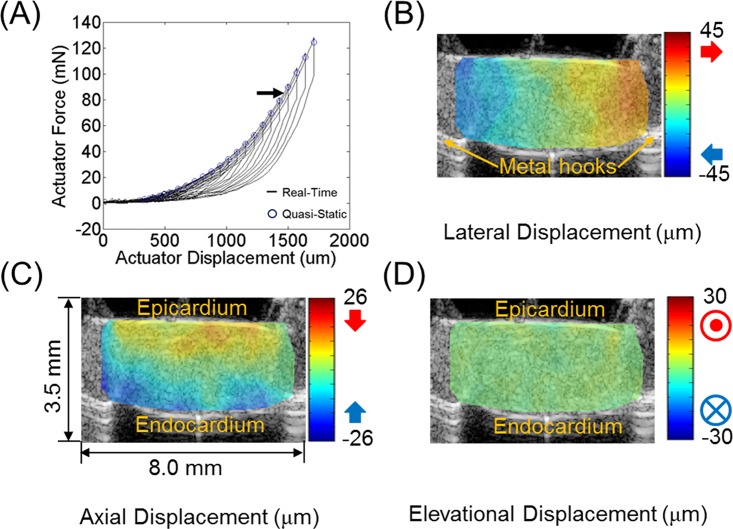
(A) Typical real time force vs. actuator displacement plot measured by the biaxial tester on right ventricular free wall (RVFW). The displacement increment between two consecutive stretch steps was 70 μm. Representative (B) lateral, (C) axial, and (D) elevational displacements estimated in the middle section (X-Z) of RVFW sample by 3D-UST between two consecutive stretch steps at actuator force of 85 mN indicated by the arrow in (A).

Fig 2A shows a typical real-time force vs. displacement plot measured by the biaxial tester on RVFW. The sample was tested with multiple loading cycles, each with an increased displacement of 70 μm and force from the previous. Fig 2B–2D depict representative displacement estimates in the middle section (X-Z) of the RVFW sample by the 3D-UST, which was obtained at actuator force of 85 mN as indicated by the arrow shown in Fig 2A. Lateral displacements (Fig 2B) show the opposite ends attached to the hooks moving away from one another, in agreement with the tensile motion imposed by the biaxial tester. Axial displacements (Fig 2C) show epi- and endo-cardium moving toward each other, resulting in compression in axial direction due to stretch in lateral direction.

**Fig 3:**
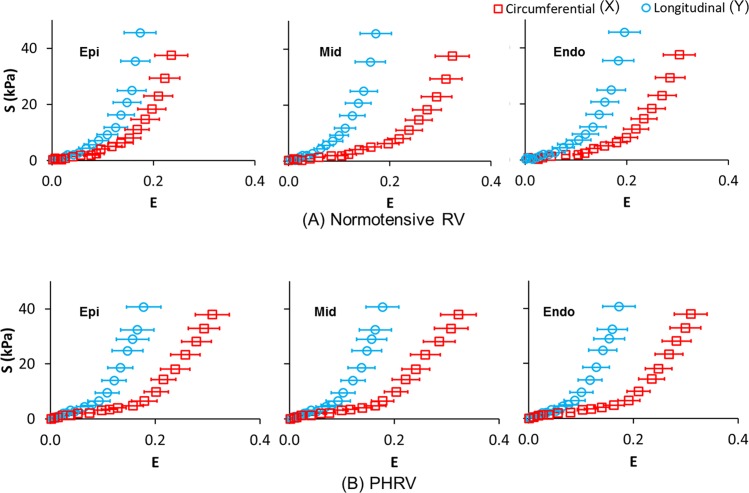
Green Lagrangian normal strain (E) obtained by 3D ultrasound speckle tracking (3D-UST) vs. second Piola-Kirchhoff normal stress (S) plots obtained by biaxial testing from epi- to endo-cardium for (A) normotensive right ventricular (RV) samples and (B) pulmonary hypertension (PH) RV samples. The error bar represents the standard deviation of average Green Lagrangian normal strains obtained from 9 normotensive RV samples and 6 PHRV samples. The normal strain (E_XX_) and normal stress (S_XX_) in circumferential direction, and normal strain (E_YY_) and normal stress (S_YY_) in longitudinal direction are presented together in the same plot for comparison.

Fig 3 presents the Green Lagrangian normal strain (E) obtained by 3D-UST vs. second Piola–Kirchhoff normal stress (S) obtained by biaxial testing plots at epi-cardium, in the middle myocardium and endo-cardium. The normal strain (E_XX_) and normal stress (S_XX_) in circumferential direction, and the normal strain (E_YY_) and normal stress (S_YY_) in longitudinal direction are presented together in the same plot for comparison. The average Green Lagrangian normal strains were obtained from 9 normotensive RV samples (Fig 3A) and 6 PHRV samples (Fig 3B). The average Green Lagrangian normal strains in circumferential direction (cross-preferred direction) and longitudinal direction (apex-to-outflow-tract direction) were marked open red squares and open blue circles, respectively. For the normotensive RV samples, the normal strain difference between the longitudinal direction and circumferential direction varied from epi- to endo-cardium (Fig 3A). The difference in normal strains increased from epi-cardium to middle myocardium, and then decreased slightly from middle myocardium to endo-cardium. On the other hand, the difference in normal strains between the longitudinal direction and circumferential direction remained similar from epi- to endo-cardium for the PHRV samples (Fig 3B).

The average SEF coefficients for 9 normotensive RV samples and 6 PHRV samples were summarized in Table 1.

**Table 1:** The average SEF coefficients for normotensive RV samples and PHRV samples.

SEF coefficient (KPa)	Normotensive RV	PHRV
C_1_	4.1 ± 0.8	25.4 ± 17.6
C_2_	95.3 ± 22.5	216.2 ± 24.4
C_3_	0.3 ± 0.3	0.3 ± 0.3
C_4_	-19.1 ± 6.2	-2.4 ± 4.1
C_5_	25.3 ± 9.9	3.6 ± 1.9

**Fig 4:**
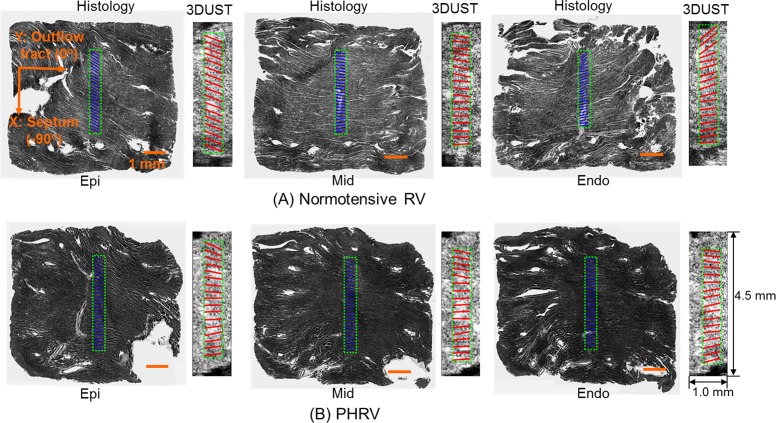
Representative reconstructed fiber orientations of (A) normotensive and (B) PHRV samples. The fiber orientation angle was defined as Y: outflow tract (0°) and X: septum (-90°). The fiber orientations obtained by 3D-UST (red solid lines) overlaid with ultrasound images compared with histology-quantified fiber orientations (solid blue lines) overlaid with corresponding histological images at near the epi-cardium, middle myocardium and near the endo-cardium.

The reconstructed fiber orientations of RVFW sample from 3D-UST-biaxial experiments were compared with histology as presented in Fig 4. Three representative histological images overlaid with histology-quantified fiber orientations in the middle areas (blue solid lines) are presented, next to the fiber orientations reconstructed (red solid lines) from 3D-UST-biaxial data overlaid with corresponding ultrasound images. These were taken from the outer (near epi-cardium), middle (middle myocardium) and inner (near endo-cardium) layers of RVFW samples. Note that the fiber orientation angle was defined as presented in Fig 4A: outflow tract (0°) and septum (-90°). The fiber orientations were averaged in areas marked green dotted lines for each axial layer. For normotensive RVFW samples, the fiber orientations distinctively changed from epi-cardium to endo-cardium (Fig 4A). On the other hand, the changes in the fiber orientations of PHRV samples from epi-cardium to endo-cardium were not noticeable (Fig 4B).

**Fig 5:**
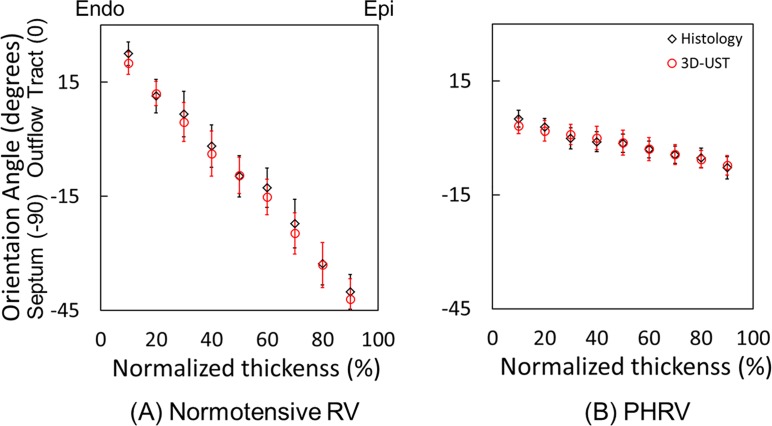
The average fiber angles computed by 3D ultrasound speckle tracking (3D-UST)-biaxial experiment, and those quantified by histology from epi- to endo-cardium for (A) normotensive right ventricular free wall (RVFW) samples and (B) pulmonary hypertension right ventricular (PHRV) samples. The error bar represents the standard deviation of average fiber angles obtained from 9 normotensive right ventricular samples and 6 PHRV samples.

Fig 5 plots the average fiber orientation angles in each 9 slices from endo- to epi-cardium of (A) 9 normotensive RVFW samples and (B) 6 PHRV samples. The average fiber orientations obtained by 3D-UST biaxial experiments and histology were represented as open red circles and open black diamonds, respectively. The fiber orientations obtained by 3D-UST biaxial experiments were not significantly different from the fiber orientations obtained by histology (P>0.05). For normotensive RVFW samples, the average fiber orientation angles obtained by 3D-UST with biaxial test sharply decreased from 20°±2.9° to -42°±5.3° (Δ = 62°±8.2°) from endo-cardium to epi-cardium. For PHRV samples, the average fiber orientation angles obtained by 3D-UST with biaxial test gradually decreased from 3°±2.0° to -7°±2.6° (Δ = 10°±4.6°) across tissue thickness. The differences in fiber orientation angles were significant between the normotensive RVFW and PHRV samples both at the epi- and endo-cardium (P<0.05). The transmural change in the fiber orientation of PHRV samples was found to be significantly smaller than that of the normotensive RVFW samples (P<0.05).

**Fig 6:**
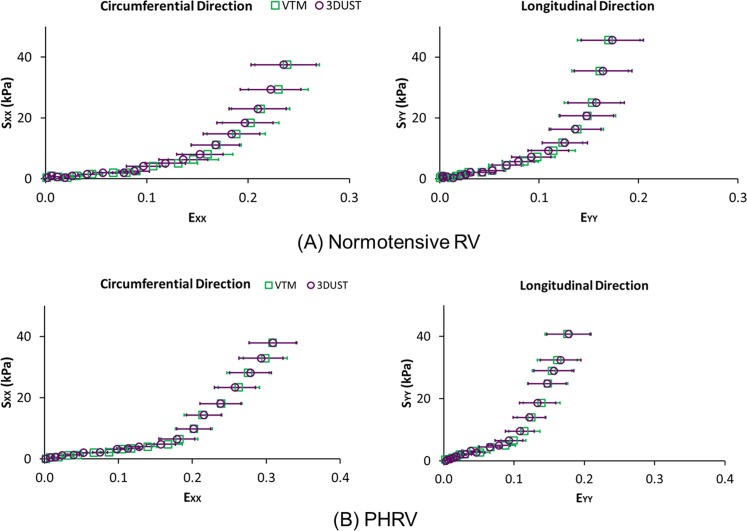
Green Lagrangian normal strain (E) vs. second Piola-Kirchhoff normal stress (S) plots measured from (A) normotensive right ventricular (RV) and (B) pulmonary hypertension RV (PHRV) samples for visually tracking markers (VTM) and 3D ultrasound speckle tracking (3D-UST). The error bar represents the standard deviation of average Green Lagrangian normal strains.

In Fig 6, Green Lagrangian normal strains (E_XX_ and E_YY_) measured by VTM and 3D-UST from (A) normotensive RVFW and (B) PHRV samples were plotted against second Piola-Kirchhoff normal stresses (S_XX_ and S_YY_) measured by the biaxial tester. The average Green Lagrangian normal strains (E_XX_ and E_YY_) obtained by VTM and 3D-UST are indicated as open green squares and open purple circles, respectively. The differences in normal strains between VTM and 3D-UST at each stress level were not significant for both circumferential and longitudinal directions (P>0.05).

**Fig 7:**
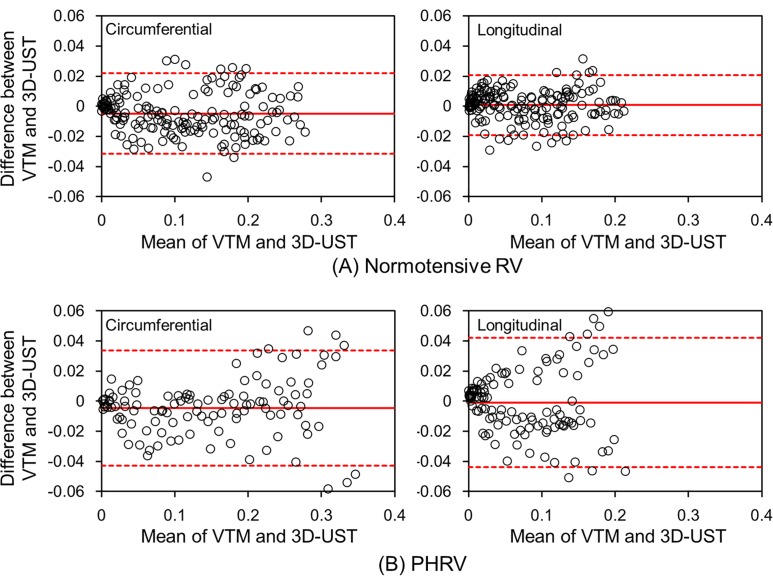
The Bland-Altman plots showing the differences of normal strains between the VTM and 3D-UST. The middle red solid line indicates the mean difference of normal strains and the upper and lower red dotted lines represent 95% limit of agreement for the two measurements.

Fig 7 presents the Bland-Altman plots showing the differences of normal strains between the VTM and 3D-UST. The middle red solid line indicates the mean difference of normal strains and the upper and lower red dotted lines represent 95% limit of agreement for the two measurements. The Bland-Altman plots illustrates that the normal strains obtained by VTM compared well to those obtained by 3D-UST without bias.

## Discussion

The fiber orientations obtained by the biaxial testing with 3D-UST matched well with the fiber orientations computed from the histology as presented in Fig 5. In addition, the stress-strain relations of the myocardium in the epi-cardial surface obtained by the biaxial testing with 3D-UST compared well with the biaxial testing with VTM for both longitudinal and circumferential directions as presented in Fig 6. These provided further validation for our technique of concurrently measuring the mechanical properties and fiber orientation with biaxial mechanical testing and 3D-UST.

The overall trends of the mechanical response to stretch at epi-cardium between normotensive RV tissue and PHRV tissue were distinctively different as shown in Fig 3. For normotensive RV tissue, the differences in Green Lagrangian normal strains between longitudinal and circumferential direction were small at epi-cardium, while they became distinctively higher at middle myocardium and endo-cardium (Fig 3A). On the contrary, for PHRV tissue, the normal strains in longitudinal direction exhibited consistently much smaller than circumferential direction at each stress level through the entire wall of RV tissue (Fig 3B). This is most likely associated with the transmural fiber orientation changes.

The fiber orientations between normotensive RVFW and PHRV samples were distinctively different as shown in Figs 4 and 5. For normotensive RVFW samples, the angle of fiber orientations sharply decreased from endo- to epi-cardium (Fig 4A) and this represents the helical clockwise rotation of transmural fiber orientation as presented by previous investigators [12, 20]. The helical orientation of fibers in myocardium is critical for ejection and suction because it provides effective torsion to deliver a stroke volume at small increases of filling pressure [21]. However, for PHRV tissue, the transmural fiber orientation lost its helical through-wall variation (Fig 4B). The loss of the transmural fiber orientation confirms prior reports [6] and is indicative of an increase of overall RV tissue anisotropy caused by PA banding [12]. The loss of helical fiber orientation reduces contractile force in RV and this can cause heart failure in ischemic and dilated cardiomyopathy [21].

The stress-strain relationship changes from epi-cardium to endo-cardium (Fig 3) should be closely related to fiber orientations in RV tissue [8] such that the mechanical stiffness of RV tissue is maximal when the fibers are aligned with the force direction [9]. Thus, differences in the stress-strain relationship between longitudinal and circumferential directions were greatest at mid-myocardium for normotensive RVFW samples since the fibers were aligned to the longitudinal direction. This difference was smallest at epi-cardium since the fibers were aligned to about -45° with respect to the longitudinal direction (Fig 5A). For PHRV samples, the fiber orientations changes from epi- through endo-cardium were small as shown in Fig 5B and the differences in stress-strain relationship were also small as presented in Fig 3B. The remodeling of fiber alignment toward the outflow tract in PHRV might be the response of RV to adapt the hypertensive ejection force. For clinical aspect, longitudinal shortening of the myocardium is a greater contributor to RV stroke volume than circumferential shortening during normal contraction [22]. The regional myocardial shortening during contraction is related with local transmural myocardial fiber orientations [23]. Therefore, the longitudinally re-oriented myofibers may reduce the longitudinal shortening.

The SEF coefficients of normotensive RV, as outlined in Table 1, had a matching order of the same magnitude as the previously reported SEF coefficients of normotensive RV [24]. However, this interpretation should be cautious since the SEF coefficients were obtained from different animal samples (dog) and the testing protocols were different. This previous study has been chosen to compare our results because there is no report of small animal biaxial RV testing using these specific forms of SEFs.

The mechanical properties and fiber orientations in RV tissues computed using the 3DUST are considered as both myofibers and collagen fibers. For the mechanical properties, both of the myofibers and collagen fibers are the dominant mechanical constituents comprising the myocardium [12]. The response of myofibers is dominant in low strains, but contributions of collagen fibers become overwhelming in large strains because the collagen is gradually recruited at higher strains to bear load. In previous study, the myofibers and collagen fibers have been observed to have similar orientations by histology [12]. Thus, in this study, the fiber orientations represent the morphology combined between myofibers and collagen fibers.

There are several limitations in our fiber orientation computation using the 3D-UST-biaxial testing approach. Firstly, we assumed the fiber orientation was purely in the lateral-axial plane (in-line with the biaxial testing) and it has no axial component. This idealization was adopted to simplify the non-linear curve-fitting computation. To fully account for 3D fiber orientation, the axial component should be included. In future studies, the computation of 3D fiber orientation may be feasible by applying a more efficient non-linear curve-fitting algorithm which reduces computation time for fiber orientations. Secondly, our present methodology cannot account for stress non-uniformity in the sample, and may thus lead to some errors. We assumed uniform stresses within a cross-section in the elevational and lateral directions. This assumption is sometimes violated since in a non-homogeneous sample such as the myocardium, spatially varying fiber orientations might cause stress non-uniformity as the sample is being stretched. Thus, further investigation of non-uniform stress distribution effect on fiber orientation computation for the non-homogeneous tissue is necessary. Thirdly, this study computed the mechanical property modelled with a simplified SEF by assuming transversely isotropy and using a simplified SEF from previous work [19]. The myocardium tissue is a complex orthotropic material and the simplified SEF may not fully characterize the mechanical property of myocardium [25]. The mechanical shear and compression tests are necessary to determine the orthotropic response of myocardium in addition to the biaxial test. The 3D-UST can apply to the shear and compression test to characterize the myocardium tissue properties. In future study, we would perform the shear and compression tests and the orthotropic myocardium responses may be computed using the SEF model without simplifications. Finally, the tissue displacement and cross-sectional areas were computed using the ultrasound signals based on the average sound wave speed in tissues, 1540 m/s. The sound wave speed in cardiac tissue should be little higher, 1570 m/s [26] and this difference of sound wave speed may cause some errors for the strain and stress calculation, but less than 2%. In future study, we would investigate the effect of sound wave speed changes on the computation of mechanical properties and fiber orientation. With the necessary modifications of fiber orientation reconstruction algorithm and/or experimental procedures if needed, this methodology can apply to other tissue samples and facilitate systematic approach for assessing and understanding better coupled structural and mechanical tissue property changes.

## Conclusions

Our results suggest that combining 3D-UST with biaxial mechanical testing may concurrently retrieve both mechanical properties and spatially-varying fiber orientations in RVFW myocardium. It may also provide 3D transmural tissue level information specific to adaptation and dysfunction of RV myocardium associated with PH. This methodology will allow for systematic approaches that might augment understanding of underlying mechanisms of RV adaptation to the increased afterload and dysfunction caused by PH, potentially leading to important new treatments in the clinic.

## Supporting Information

S1 File The animal research procedures and welfare considerations. (PDF)
