# Supplementary material for: Quantification of Coupled Stiffness and Fiber Orientation Remodeling in Hypertensive Rat Right-Ventricular Myocardium Using 3D Ultrasound Speckle Tracking with Biaxial Testing
Source: PLoS One. 2016 Oct 25;11(10):e0165320. doi: 10.1371/journal.pone.0165320 (PMC5079565; doi:10.1371/journal.pone.0165320)
Supplement: S1 File — (PDF) [file pone.0165320.s001.pdf]

The animal research procedures and welfare considerations

The experimental protocol of this study was approved by the University of Pittsburgh Institutional Animal Care and Use Committees. A total of 15 male Sprague–Dawley rats, 8 weeks old at the start of the experiment, were used in this study. Of the 15 total rats, RV pressure overload was induced surgically by restriction of the pulmonary artery (PA) on 6 animals. The PA banding was kept for 3 weeks for all 6 animals to generate a chronic pressure overload. The PA bending was performed as following. Animals were anesthetized with 5% isoflurane and placed on a heated table to maintain a core temperature of 37˚C, then a lateral thoracotomy was performed and a surgical clip was placed around the PA to generate a uniform RV pressure of 45–50 mmHg (approximately the diameter of a 27-gauge needle). After chest closure, the animals were extubated and observed for 2 hours. The animals were then monitored per standard practice for 3 weeks. A total of 8 animals died during the PA banding surgery, while they were still under anesthesia and therefore had no symptoms. All animals were treated humanely as directed by the University of Pittsburgh Institutional Animal Care and Use Committee, with procedures in place to treat symptoms.
